# *Thelazia lacrymalis* in horses from Romania: epidemiology, morphology and phylogenetic analysis

**DOI:** 10.1186/s13071-022-05532-z

**Published:** 2022-11-14

**Authors:** Vlad-Dan Cotuțiu, Angela Monica Ionică, Menelaos Lefkaditis, Cristina Daniela Cazan, Alina Diana Hașaș, Andrei Daniel Mihalca

**Affiliations:** 1 Department of Parasitology and Parasitic Diseases, University of Agricultural Sciences and Veterinary Medicine of Cluj-Napoca, Calea Mănăștur 3-5, 400372 Cluj-Napoca, Romania; 2 CDS-9, University of Agricultural Sciences and Veterinary Medicine of Cluj-Napoca, Calea Mănăștur 3-5, 400372 Cluj-Napoca, Romania; 3 Clinical Hospital of Infectious Diseases of Cluj-Napoca, Iuliu Moldovan Street no. 23, Cluj-Napoca, Romania; 4 Laboratory of Microbiology and Parasitology, Department of Veterinary Medicine, School of Health Sciences, University of Thessaly, 43100 Karditsa, Greece; 5 Department of Physiopathology, University of Agricultural Sciences and Veterinary Medicine of Cluj-Napoca, Calea Mănăștur 3-5, 400372 Cluj-Napoca, Romania

**Keywords:** *Thelazia lacrymalis*, Equine thelaziosis, Romania

## Abstract

**Background:**

Equine thelaziosis is a neglected vector-borne parasitic disease in modern veterinary medicine, lacking recent reports. It is transmitted by *Musca autumnalis*, and potentially other Muscidae species, by ingesting the lachrymal secretions of its equine host. The distribution of both *Thelazia lacrymalis* and its intermediate hosts remains largely unknown throughout Europe, with most studies dating back 20 years. The aim of this study was to assess the presence, prevalence and distribution of *T. lacrymalis* in horses from Romania.

**Methods:**

The eyes of 273 horses, slaughtered at two abattoirs from the Northwestern and Western regions of Romania, were examined for the presence of *T. lacrymalis* between March and November 2021. Upon detection, the nematodes were collected and morphologically identified using the keys from literature. Following identification, one specimen from each animal was selected for molecular analysis while the rest underwent detailed morphometric measurements. Mapping and distribution, according to ecoregions, was done using the QGis 3.20 software, while sequences obtained were compared to those available in GenBank through BLAST analysis using the MEGA X software.

**Results:**

Of the 273 animals sampled, 12 (4.39%) were positive for *Thelazia* spp. infection. Eighty-seven nematodes were recovered, all morphologically identified as *T. lacrymalis*. The intensity of infestation varied between one and 33 nematodes/animal while five animals presented a bilateral infestation and seven a unilateral one. The highest prevalence was encountered in Pannonian ecoregion (12.12%) while the lowest was in the Alpine ecoregion (0%). Seventy-five intact specimens underwent detailed morphometric analysis, of the 18–20 parameters, resulting in notable differences in striation lengths compared to the data available in other reports. BLAST analysis identified a 96.46–98.60% similarity to the only other *COI* gene sequence available for *T. lacrymalis*.

**Conclusions:**

The current study represents the first report of *T. lacrymalis* in horses in Romania. The low prevalence rates are probably linked to the wide use of macrocyclic lactones.

**Graphic Abstract:**

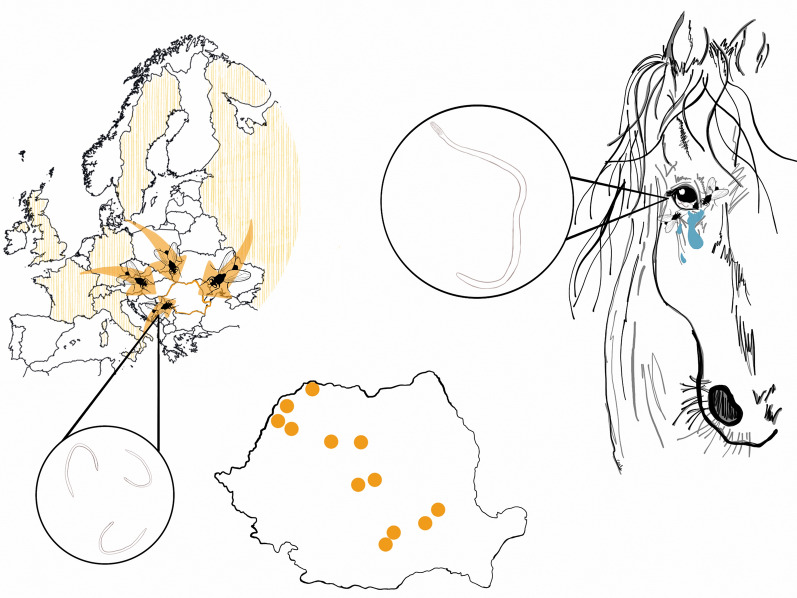

## Background

Thelaziosis is a parasitic disease caused by nematodes of genus *Thelazia* in the conjunctival sack of their hosts. The intermediate hosts for various *Thelazia* species are non-biting secretophagous flies, which ingest the first stage larvae during their meal on lachrymal secretions [1]. For *Thelazia* spp. infecting large ruminants and horses, *Musca autumnalis* seems to be the main vector. *Musca domestica* as well as other Muscidae species have also been suggested as vectors [2, 3]. The disease has garnered much attention within the past 2 decades, following the emergence of *Thelazia callipaeda* in carnivores and other hosts, including humans, throughout much of Europe [4]. Additionally, occasional reports of cases in large ruminants still emerge from time to time in Europe [5, 6]. In Romania, *Thelazia* spp. were so far found in domestic and wild carnivores [7–10] and cattle [6, 11].

In horses, the disease is poorly studied, and its epidemiology remains largely unknown. The only species reported in horses is *Thelazia lacrymalis*, first described in Germany in the nineteenth century [12]. The species is cosmopolitan, ranging from Asia to the Americas and Europe [3, 13–19]. However, reports of thelaziosis in horses in Europe are scarce, with the last published case in 2007 in Germany [20]. With the exception of the former USSR, where equine thelaziosis has been reported in the region of Bashkortostan (at the very eastern limit of geographical Europe) [3], equine thelaziosis has not been documented in Eastern Europe. One report of *T. lacrymalis* was published from Switzerland in horses imported from Poland and Hungary, but the infection site of horses is not identified with certainty [2].

The aim of our study was to investigate the occurrence of *Thelazia* spp. in horses from Romania. Additionally, we provide detailed morphometric data to improve the species description.

## Materials and methods

Samples consisted of both eyes belonging to 273 horses slaughtered at two different abattoirs from Northwestern and Western Romania between March and December 2021 (Table 1). For each horse, the following data were collected: sampling date, age, sex and origin (locality, geographic coordinates, altitude and ecoregion).

**Table 1:** Sampled horses according to their age category, altitude interval, sex and ecoregion

Variable		Sampled	Positive	Prevalence (%)	95% CI
Sex	Males	136	5	3.67	1.58–8.32
	Females	137	7	5.11	2.5–10.17
Age interval (months)	1–131	81	7	8.64	4.25–16.78
	132–251	125	4	3.2	1.25–7.94
	252–371	60	1	1.66	0.29–8.86
	≥ 372	7	0	0	0–35.43
Altitude interval (meters)	0–100	38	0	0	0–9.18
	101–200	73	6	8.22	3.82–16.79
	201–300	50	2	4	1.1–13.46
	301–400	58	2	3.44	0.95–11.73
	401–500	23	2	8.69	2.42–26.8
	≥ 501	30	0	0	0–11.35
Ecoregion	Pannonian	33	4	12.12	4.82–27.33
	Continental	161	7	4.35	2.12–8.7
	Alpine	45	0	0	0–7.87
	Steppic	34	1	2.94	0.52–14.92
Total		273	12	4.39	2.53–7.52

In the slaughterhouse, the eyes of each animal were removed along with adjacent tissues, namely eyelids and lachrymal glands, without perforating the conjunctival sack and individually placed in a sealed zip bag. Any visible *Thelazia* worms were collected in a 1.5-ml plastic tube with saline and placed in the same zip bag as the eyes they belonged to. All samples were then transported to the Department of Parasitology and Parasitic Diseases of the University of Agricultural Sciences and Veterinary Medicine of Cluj-Napoca for detailed examination. Upon arrival, the samples were transferred to a refrigerator prior to examination, which was done in maximum 48 h.

Each eye and adjacent structures were carefully examined by opening the lateral canthus followed by the eversion of the eye globe. The third eyelid was inverted and partially detached allowing the lachrymal ducts to be dissected. Subsequently, each eye was flushed with physiological saline along with their corresponding bag into a Petri dish. The content of the Petri dish was examined under a zoom stereomicroscope. All collected nematodes were placed in vials with physiological saline and kept in a refrigerator until morphological identification.

Each nematode was morphologically identified to species and developmental stage based on morphological keys described in literature [3, 16, 21]. The undestroyed specimens were preserved in 4% formalin solution and processed by detailed morphometric analysis including 19 parameters in adult males and larvae and 20 in adult females, as shown in Table 3. Morphological identification and measurements were carried out using the Olympus microscope (Olympus BX61) and dedicated software. The number of nematodes was independently recorded for each animal, by stage and sex.

One nematode from each horse was randomly selected and stored in 70% ethanol for further molecular characterization. DNA was extracted individually from 12 nematodes using ISOLATE II Genomic DNA Kit (Bioline Meridian Bioscience, Luckenwalde, Germany), according to the manufacturer’s instructions, and stored at − 20 °C until further use. A PCR amplification targeting the mitochondrial cytochrome oxidase I *(COI)* gene region (670 bp) was performed in 25 μl reaction volume, containing 12.5 μl My Taq^®^ Red PCR Mastermix (Bioline Meridian Bioscience, Luckenwalde, Germany), 6.5 μl of ultrapure water, 1 μl (10 pmol) of each of the two previously described primers [22], COIintF 5′-TGATTGGTGGTTTTGGTAA-3′ and COIintR 5′-ATAAGTACGAGTATCAATATC-3′, and 4 μl aliquot of isolated DNA. One negative control (PCR water) was included. The PCR was performed using a C1000™ Thermal Cycler (Bio-Rad, London, UK), with the following conditions: initial denaturation at 95 °C for 5 min, followed by 40 cycles of denaturation at 95 °C for 45 s and annealing at 47 °C for 45 min with extension at 72 °C for 1 min. A final extension at 72 °C for 5 min was performed. Amplification products were visualized by electrophoresis on 1.5% agarose gel stained with ECO Safe 20,000 × Nucleic Acid Staining Solution (Pacific Image Electronics, New Taipei, Taiwan), and their molecular weight was assessed by comparison to a molecular marker (HyperLadder™ 100 bp, Bioline Meridian Bioscience, Luckenwalde, Germany). The quality of the samples was visually assessed via gel electrophoresis before samples underwent purification. All PCR products were purified using the ISOLATE II PCR and Gel Kit (Bioline Meridian Bioscience, Luckenwalde, Germany) and sent for sequencing in both directions (Macrogen Europe, Amsterdam, The Netherlands). The attained chromatograms were assembled, and consensus sequences were edited and translated to corresponding proteins using Geneious 4.8.5 software (Biomatters Ltd., Auckland, New Zealand). The consensus sequences were compared to those available in the GenBank^®^ database by means of Basic Local Alignment Search Tool (BLAST).

The statistical analysis was performed using EpiInfo™ 7 software (CDC, USA). The frequency prevalence and 95% confidence interval (CI) of infestation were calculated both overall and according to various categories (Table 1). The differences among categories were assessed by means of Chi-square testing. Mapping and distribution were carried out using the QGis software (version 3.20).

The evolutionary analyses were conducted using MEGA X software [23]. The analysis involved 18 nucleotide sequences: 12 attained within the present study, five sequences of *Thelazia* spp. retrieved from GenBank and one *Dirofilaria immitis* sequence as outgroup. The sequences were aligned using the MUSCLE algorithm, and the evolutionary history was inferred by using the maximum likelihood method and Tamura-Nei model [24]. A discrete gamma distribution was used to model evolutionary rate differences among sites.

## Results

**Fig. 1:**
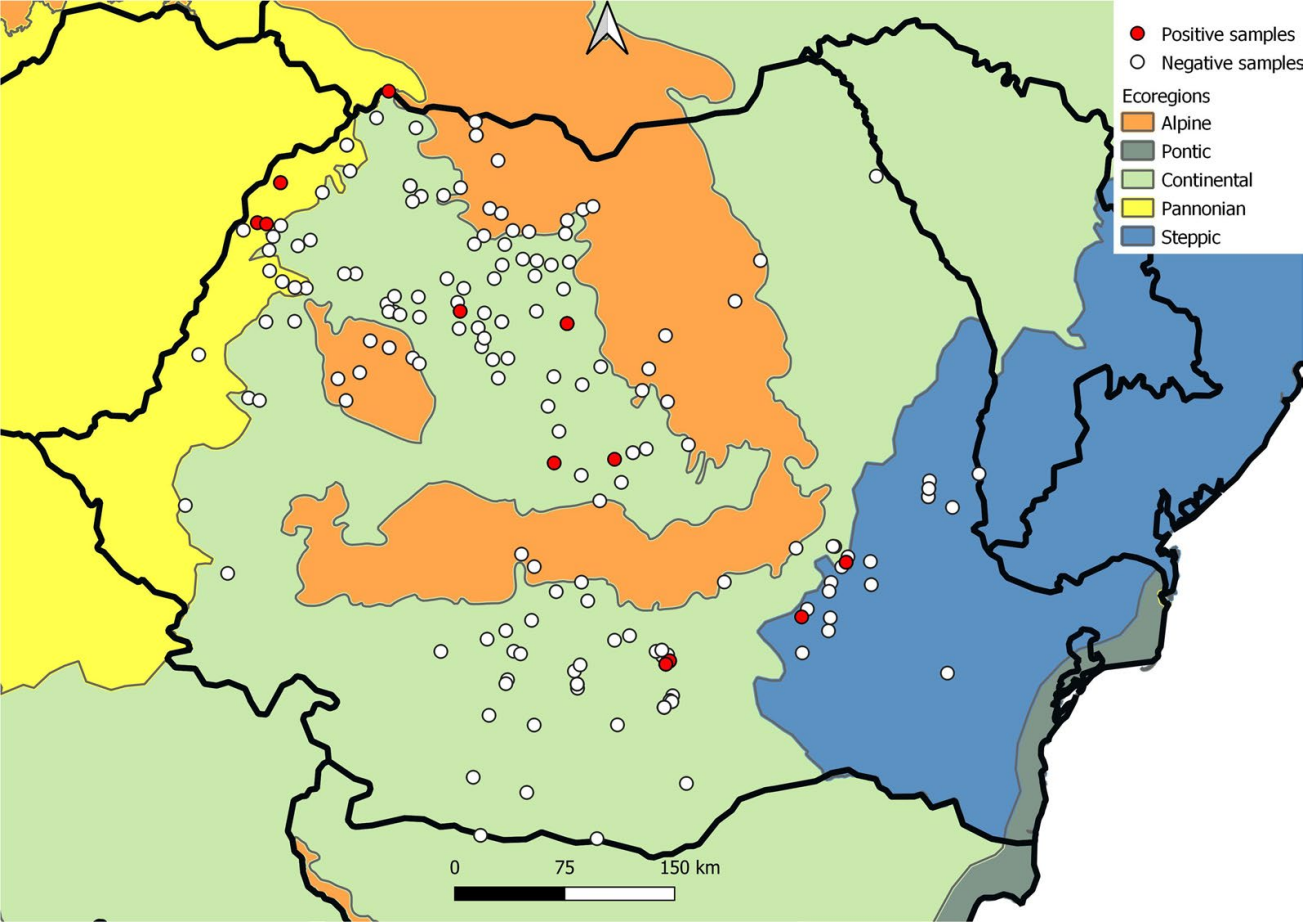
Distribution of *T. lacrymalis* in horses from Romania shown by ecoregion

Of the 273 animals sampled, 12 (4.39%) were positive for *Thelazia* spp. infestation (Table 1). A total of 87 nematodes were collected (Table 2). Of the infected animals, five presented a bilateral infestation (41.66%) while the rest had a unilateral one (58.33%). The intensity varied between one and 33 nematodes per animal (mean intensity 7.25 nematodes/infected horse and median of 4) with an intensity between one and 20 nematodes/infested eye (mean intensity of 2.66 and 4.58 nematodes per eye, respectively). The adult female-to-male ratio was 3.4 to 1. All individuals were morphologically identified as adults and larvae (L5) of *T. lacrymalis*. There were no statistically significant differences between the prevalence in any of the considered categories (sex, age group, altitude, ecoregion). Of the four ecoregions from which samples were collected, *T. lacrymalis* was found in three (Fig. 1).

**Table 2:** Population structure of *Thelazia lacrymalis* in horses in Romania

Code	Total	Males	Females			Laterality
		Adults	L5	Adults	Total	
BHC1-1	31	5	10	16	26	Bilateral
MMC1-21	11	4	0	7	7	Bilateral
MMC1-44	2	1	0	1	1	Unilateral
MMC1-14	3	0	0	3	3	Unilateral
BHC1-4	7	3	1	3	4	Bilateral
BHC2-11	4	1	2	1	3	Unilateral
BHC3-12	5	0	2	3	5	Unilateral
BHC3-31	4	2	0	2	2	Unilateral
BHC3-7	1	0	0	1	1	Unilateral
BHC4-12	4	1	2	3	3	Bilateral
BHC4-49	3	0	2	1	3	Unilateral
BHC4-46	10	1	3	6	9	Bilateral
Total	85	18	22	45	67

**Fig. 2:**
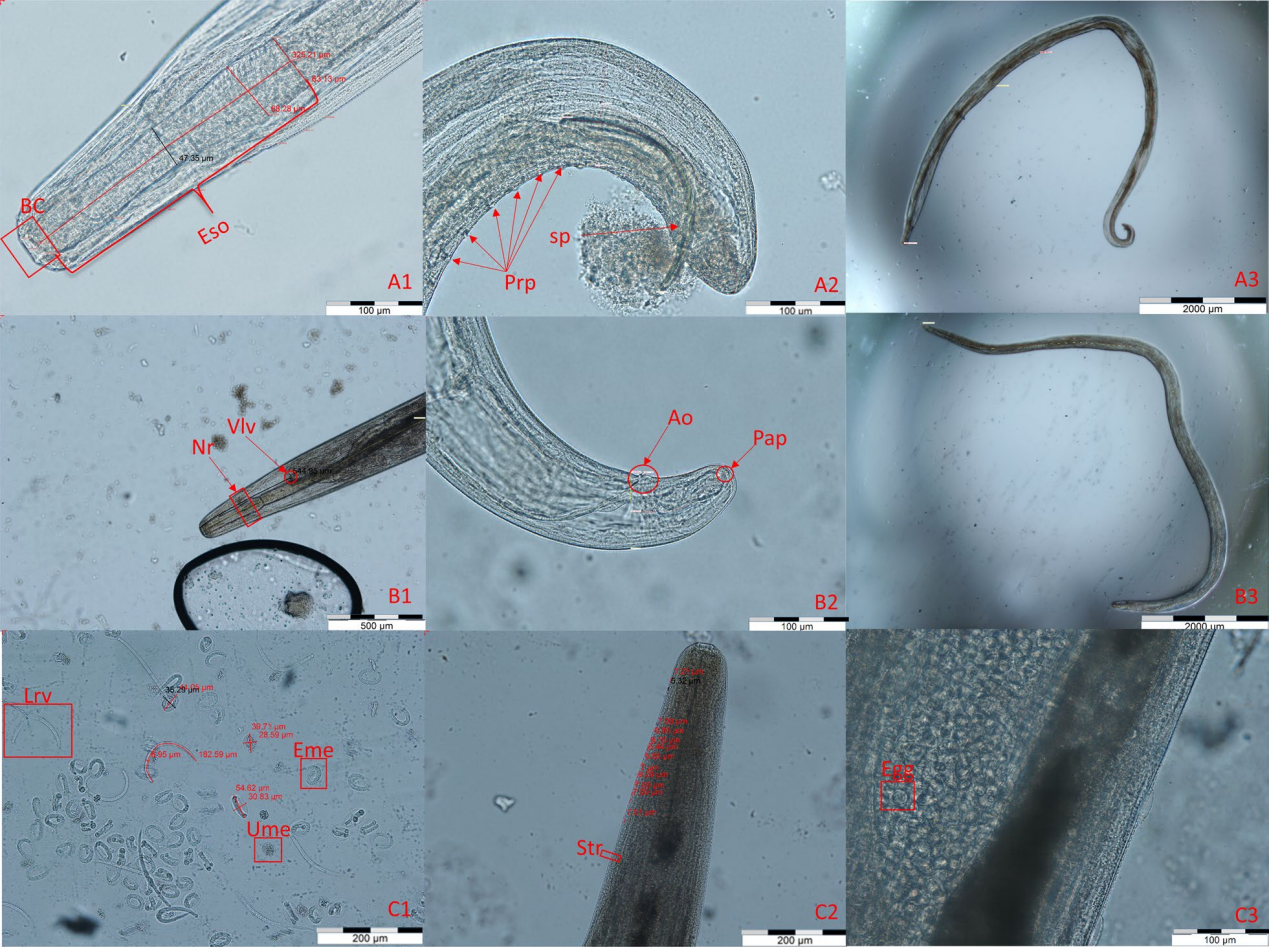
Morphological features of *T. lacrymalis*. A1: Cervical region of a *T. lacrymalis* male; A2: tail of a *T. lacrymalis* male; A3: total length of a *T. lacrymalis* male; B1: cervical region of a *T. lacrymalis* female, with the nerve ring (Nr) positioned in the lower third of esophagus and the vulvar opening (Vlv) positioned distally from esophago-intestinal junction; B2: tail of a *T. lacrymalis* female; C1: contents of ruptured uterus of a gravid *T. lacrymalis* female with measurements; C2: cervical striations in a *T. lacrymalis* female with measurements; C3: gravid *T. lacrymalis* female with a highlighted egg. Abbreviations: BC, buccal capsule; Eso, esophagus; Sp, spicule; Prp, pre-anal papillae; Ao, anal orifice; Pap, post-anal papillae; Lrv, larvae; Eme, eggs containing larvae; Ume: blastomerized eggs; Str, striations

Of the 85 *T. lacrymalis* specimens, 75 were selected for a detailed morphometric analysis (Fig. 2) (16 adult males, 36 adult females, 22 female L5). The results are shown in Table 3.

**Table 3:** Morphometric analysis of *T. lacrymalis* collected from horses in Romania (n = 75) and comparison with available data from other reports

Parameter		Males				Females					
		Adults				L5		Adults			
		CS^a^	Bz^b^	Nm^c^	Skrj^d^	CS^a^	CS^a^		Skrj^d^	Bz^b^	Nm^c^
Length		6514.56–9597.44 (7943.98)	6200	–	5696	6262.67–12,371 (9485.84)	7588.73–17,299.9 (11,948.05)		5696–18,000	10,500	12,500
Width	Anterior	98.88–144.27 (118.05)	–	–	91.75–172.6 (122.67)	105.15–189.78 (133.21)	105.15–189.78 (133.21)		–	–	–
	Median	196.76–289.84 (252.64)	237	–	181.64–336.91 (261.67)	220.13–415.59 (275.81)	220.13–415.59 (275.81)		208	289	279
	Tail	62.48–131.27 (93.42)	–	–	65.14–112.91 (86.29)	72.46–117.08 (93.52)	72.46–117.08 (93.52)		–	–	–
Buccal capsule	Proximal width	18.95–29.17 (27.57)	26	–	20.22–41.03 (28.66)	21.78–34.9 (28.23)	21.78–34.9 (28.23)		–	36	35
	Distal width	13.15–28.18 (24.43)	26	–	20.23–37.63 (25.68)	19.43–39.64 (25.94)	19.43–39.64 (25.94)		–	31	–
	Depth	10.26–17.98 (15.84)	10	–	11.43–20.1 (16.6)	12.4–20.75 (16.45)	12.4–20.75 (16.45)			21	–
Nerve ring position	Anterior distance	189.17–256.45 (220.75)	187	–	201.72–263.09 (232.76)	202.66–322.38 (236.11)	202.66–322.38 (236.11)		192–208	218	–
	Posterior distance	56.29–139.69 (90.09)	–	–	76.15–180.33 (115.34)	50.18–105.27 (105.27)	50.18–105.27 (105.27)		–	–	–
Esophagus	Length	292.18–350.15 (319.84)	312	–	287.01–443.42 (344.03)	309.05–411.1 (340.06)	309.05–411.1 (340.06)		335–352	333	–
	Proximal width	34.12–51.73 (46.82)	42	–	33.83–63.82 (49.84)	39.93–60.34 (50.7)	39.93–60.34 (50.7)		48	42	–
	Median width	52.25–83.88 (69.24)		–	45.94–87.27 (69.77)	61.44–85.5 (70.37)	61.44–85.5 (70.37)		–	–	–
	Distal width	45.75–70.77 (55.46)	62	–	41.5–68.08 (57.58)	50.63–74.65 (58.86)	50.63–74.65 (58.86)		80	62	–
Striation	Anterior	4.9–7.85 (6.09)	4.56	–	3.93–7.64 (5.82)	3.69–9.09 (6.4)	3.69–9.09 (6.4)		–	4.65	4.65
	Median	5.72–8.92 (7.34)	3.48	–	3.76–7.76 (5.97)	4.95–9.77 (7.13)	4.95–9.77 (7.13)		–	3.41	3.41
	Posterior	2.69–6.77 (4.97)	3.74	–	3.17–6.18 (4.54)	3.51–7.82 (5.07)	3.51–7.82 (5.07)		–	3.62	3.62
Anal pore		74.3–160.46 (90.52)	83	–	120	75.22–127.52 (103.39)	60.46–114.55 (101.43)		–	83	–
Egg width							15.9–37.25 (26.46)		25	–	–
Egg length							12.98–28 (18.87)		120	–	–
Vulva						549.67–588.3 (566.56)	568.34–607.8 (586.83)		528–560	593	493
Spicules length		124.31–180.56/ 108.11–159.49 (149.8/137.42)	160/ 100		180/ 120						

All measurements from the current study given in µm as range (mean)

^a^Current study

^b^Beelitz et al. [16]

^c^Naem [21]

^d^Skrjabin et al. [3]

**Fig. 3:**
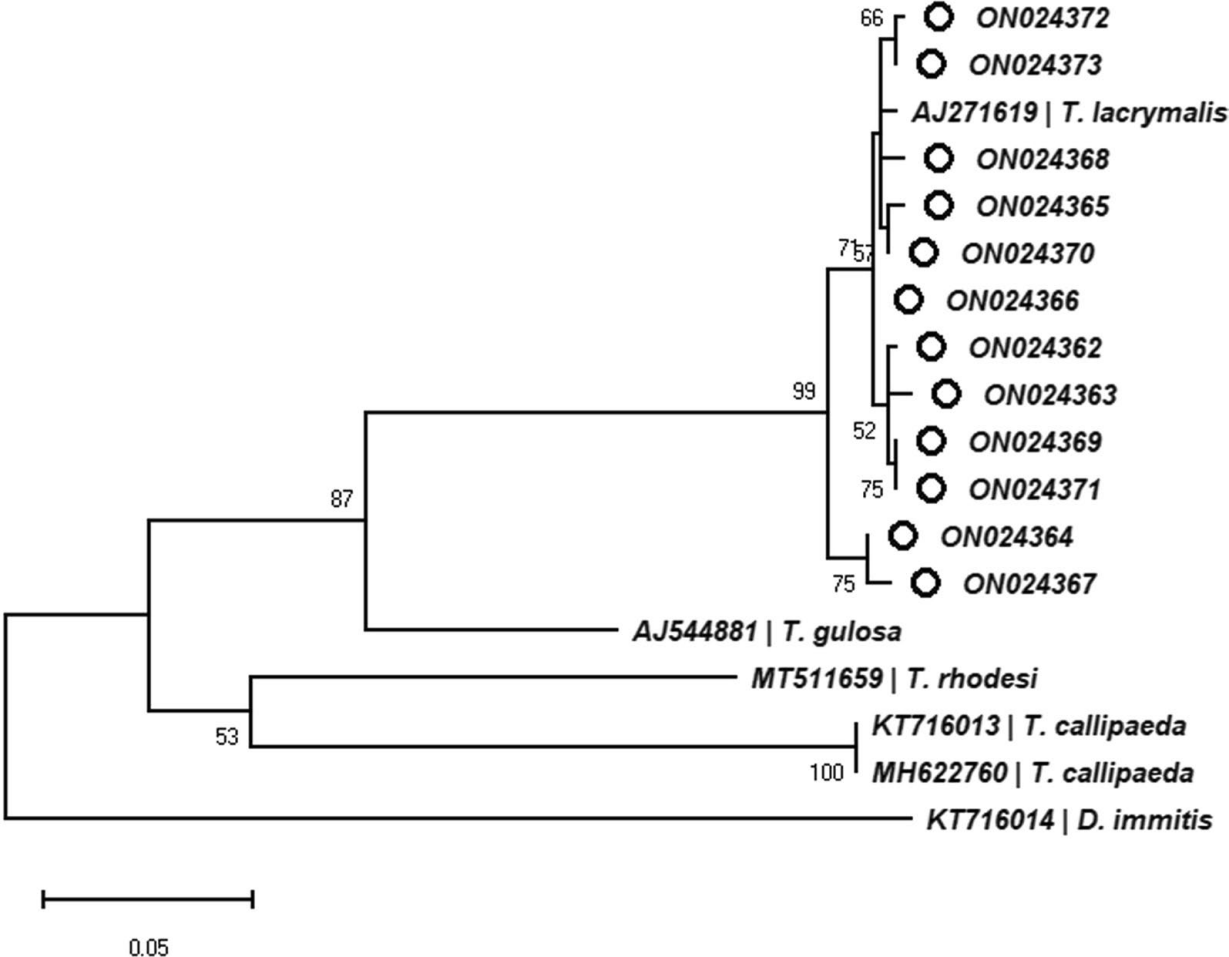
Phylogenetic tree. The bootstrap consensus tree inferred from 1000 replicates. The percentage of trees in which the associated taxa clustered together is shown next to the branches (only values > 50% are shown). The tree is drawn to scale, with branch lengths measured in the number of substitutions per site. This analysis involved 18 nucleotide sequences. There were a total of 571 positions in the final dataset

All 12 specimens selected for molecular analysis were successfully sequenced, and 11 unique sequences of the *COI* gene were obtained. The BLAST analysis revealed a 96.46–98.60% nucleotide similarity to the only *T. lacrymalis* *COI* isolate from GenBank (AJ271619). The similarity to other species of *Thelazia* (e.g. *Thelazia gulosa* AJ544881, *Thelazia rhodesi* MT511659, *T. callipaeda* AM042556) ranged between 85.05 and 88.91%. Our sequences were deposited in GenBank under the accession numbers listed in Table 4. Phylogenetic relationships are presented in Fig. 3.

**Table 4:** BLAST analysis results and accession numbers of morphologically identified *T. lacrymalis* submitted for molecular analysis

Code	Product (bp)	Molecular ID	Query (%)	Identity (%) with AJ271619	Accession no.
14MMC1	570	*T. lacrymalis*	100	96.80	ON024362
21MMC1	663	*T. lacrymalis*	97	98.15	ON024363
44MMC1	656	*T. lacrymalis*	98	96.92	ON024364
1BHC	659	*T. lacrymalis*	98	98.62	ON024365
4BHC	660	*T. lacrymalis*	98	98.92	ON024366
11BHC2	654	*T. lacrymalis*	99	96.46	ON024367
7BHC3	646	*T. lacrymalis*	99	98.75	ON024368
12BHC3	653	*T. lacrymalis*	99	98.46	ON024369
31BHC3	657	*T. lacrymalis*	98	98.92	ON024370
12BHC4	653	*T. lacrymalis*	99	98.46	ON024371
49BHC4	653	*T. lacrymalis*	99	98.92	ON024372
46BHC4	655	*T. lacrymalis*	98	98.77	ON024373

## Discussion

The lack of data over the past decades concerning the distribution of *Thelazia* in large herbivores could be attributed to the appearance of more affordable and potent anthelmintics [25] as well as the overall lack or mildness of clinical signs. Although equine thelaziosis is rarely diagnosed, it may represent an animal welfare concern due to the chronical and sometimes irreversible development of the disease or concurrent diseases [13, 26] combined with its potentially rapid spread in larger herds.

In terms of geographical distribution, equine thelaziosis has been occasionally reported in Europe in Russian Federation, England, France, Germany, Sweden and Italy [3, 14–17, 20, 27]. The current study acts as the first report of equine thelaziosis in the last 15 years in Europe, and the first from Romania and Eastern Europe, with the exceptions of old reports from the former USSR. Although the prevalence is relatively low, we consider that the lack of reports is related to the limited interest of researchers in this disease and the probably limited or absent awareness of veterinary clinicians. The low prevalence could also be attributed to applications of general oral deworming protocols used in horses, which include the use of either ivermectin or fenbendazole [28]. There were no noticeable differences in overall prevalence values during different seasons, the overall value remaining at around 5% throughout the year. Adults have been encountered during every season. L5 females were found only from July to the end of October.

During the present study, no statistically relevant results could be quantified because of the low sample number and wide distribution of both geospatial location and developmental stages. It can be however presumed that altitude is a determining factor in the occurrence of thelaziosis. In this study there were no infested animals originating from the alpine ecoregion (0 of 45); therefore, a plausible explanation could be the decreased vector abundance and shorter seasonal activity at higher altitudes [29–31].

Although clinical signs have been associated with the presence of adults, in most species, it has been theorized that the death of larvae within the lachrymal glands could be responsible for the appearance of coalescing granulomas following the administration of oxybendazole in horses [32]. This theory also underlines the importance of larvae in the pathological processes inherent to conjunctivitis, dacryocystitis and ultimately keratoconjunctivitis sicca [33].

The morphological description was done to improve the data availability for the identification of this poorly known species. Our results provide additional data on 18–20 parameters (Table 3), extending the previously documented variations, in adult parasites (both mature and immature stages) as well as comparing our findings to those available in other studies.

Despite the low number of *COI* gene sequences for nematodes of genus *Thelazia* available for the phylogenetic analysis, we showed that *T. lacrymalis* represents a separate clade within the genus.

## Conclusion

Equine thelaziosis is present in Romania at low prevalence values, related probably to the widespread use of macrocyclic lactones. We consider equine thelaziosis a neglected disease in Europe, which requires more attention from veterinary practitioners mainly from an animal welfare point of view due to the potentially severe clinical impact.

## Data Availability

All data generated or analyzed during this study are included in this published article as well as its additional data. Sequences generated in this study are available in GenBank (ON024362-ON024373).
